# Prevalence of small intestinal bacterial overgrowth in irritable bowel syndrome (IBS): Correlating H_2_ or CH_4_ production with severity of IBS

**DOI:** 10.1002/jgh3.12899

**Published:** 2023-04-03

**Authors:** Philippe Onana Ndong, Hanae Boutallaka, Eugenia Marine‐Barjoan, Dann Ouizeman, Raja Mroue, Rodolphe Anty, Geoffroy Vanbiervliet, Thierry Piche

**Affiliations:** ^1^ Gastro‐entérologie, Hôpital L'Archet 2 Centre Hospitalier Universitaire de Nice Nice France

**Keywords:** gastrointestinal disorders, glucose breath test, hydrogen, irritable bowel syndrome, methane, small intestinal bacterial overgrowth

## Abstract

**Background and Aim:**

The prevalence and the role of small intestinal bacterial overgrowth (SIBO) in irritable bowel syndrome (IBS) remain unclear, as the literature provides heterogeneous information on the subject. The aim of this study was to determine the prevalence of SIBO in IBS and to assess the correlation between methane and hydrogen levels measured during breath tests and the severity of IBS.

**Method:**

Two‐hundred and forty‐seven patients with IBS were prospectively included. A glucose breath test (GBT) measured H_2_ and CH_4_ production to diagnose SIBO. A test was positive when H_2_ values exceeded 12 ppm in the first 90 min and/or when a CH_4_ value exceeded 10 ppm at any time. IBS severity (IBS‐SSS), quality of life (GIQLI), and anxiety and depression (HAD) were assessed to investigate the correlation with H_2_ and CH_4_ production.

**Results:**

The prevalence of SIBO in IBS was 36.4% (9.7% with H_2_, 26.7% with CH_4_). CH_4_ levels were significantly higher in the predominantly constipated patients (*P* = 0.00), while H_2_ levels were significantly higher within the diarrheal phenotype (*P* = 0.01). IBS severity was not correlated with either H_2_ levels (*r* = 0.02; *P* = 0.84) or CH_4_ levels (*r* = 0.05; *P* = 0.64). H_2_ production was inversely correlated with the quality of life (*r* = −0.24; *P* = 0.03) and significantly correlated with the HAD scale (*r* = 0.22; *P* = 0.03). The pain and discomfort experienced during GBT was not correlated with methane levels (*r* = −0.09, *P* = 0.40), hydrogen levels (*r* = −0.01, *P* = 0.93), or sum of both (*r* = 0.06, *P* = 0.58), but significantly associated with IBS severity (*r* = 0.50, *P* <0.00).

**Conclusion:**

SIBO has a high prevalence in IBS but does not increase its severity. Individual susceptibility to pain may have a greater influence on the severity of IBS.

## Graphical abstract

The prevalence of SIBO in this cohort of IBS patients was 36.4%. In IBS patients who tested positive for SIBO, gas levels (hydrogen and methane) measured during glucose breath tests were not correlated with either the severity of IBS symptoms or the level of pain experienced during the test. We found significantly higher values of methan in consitpated patients while hydrogen production was significantly higher in patient with a diarrheal phenotype.

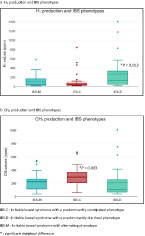

## Introduction

Irritable bowel syndrome (IBS) is the most common functional digestive disorder. In developed countries, its prevalence varies between 10% and 15%. It is mainly characterized by abdominal pain and transit disorders, meeting the Rome IV criteria.^1^ The chronic course of these symptoms can lead to a severe impairment of patients' quality of life, and hence there is need for a better understanding of the pathophysiological mechanisms and the factors that can influence the severity of this disease.

The pathophysiology of IBS is complex and may involve several elements such as intestinal hyperalgesia, dysregulation of the intestinal immune system, and micro‐inflammatory state, but also qualitative and quantitative perturbations of the intestinal microbiota.^2^ This last point is the subject of particular attention because of the frequently observed association between IBS and small intestinal bacterial overgrowth (SIBO).

SIBO is characterized by a quantitative excess of small intestinal bacteria, which are responsible for the production of gases including hydrogen and for micro‐inflammatory mechanisms that may contribute to the development of symptoms similar to those encountered in IBS. Disorders of the methanogenic flora, mainly constituted by archaebacteria, have also been associated with SIBO, although the terminology may seem less appropriate. In fact, the majority of these archaea use hydrogen from anaerobic fermentation as a substrate to produce methane.^3^ Thus, the measurement of methane during breath tests has been proposed to increase their diagnostic sensitivity concerning SIBO. The role of these gases in the development of symptoms still needs to be investigated. They could have an impact on bowel transit, since a frequent association between methane production and the development of a constipated phenotype has been demonstrated in IBS patients. Moreover, according to some authors, the severity of the constipation may also be correlated with the level of methane production.^4^ Such evidence suggests that there may be a correlation between the level of gases produced by intestinal flora and the overall severity of IBS. However, very few studies have explored this question, showing heterogeneous results. Additional data may help determine whether SIBO should be considered a factor in the severity of IBS, setting its eradication as a major therapeutic target in these patients.

In the published literature, the prevalence of SIBO in IBS varies widely between 4%^5^ and 76%.^6^ The reliability of these data is limited by the heterogeneity of the populations, the use of variable diagnostic methods, and variable positivity criteria. Breath tests are nowadays a major tool for the diagnosis of SIBO, but their performances vary according to the substrates and the positivity thresholds applied to methane and hydrogen. Concerning the choice of substrates, glucose seems to give the most reliable results, although lactulose can also be used in this situation. The European^7^ and American^8^ consensus conferences on breath test indication and interpretation have recommended some criteria, which are still being evaluated. A recent meta‐analysis, which reviewed the performance of breath tests with different positivity thresholds,^9^ has supported those recommended by both conferences for methane (>10 ppm at any time during the breath test). However, regarding hydrogen production, the results of this meta‐analysis suggest that a threshold of 12 ppm should be preferred over the 20‐ppm threshold recommended by the American conference. These criteria are expected to provide a solid framework for more reliable studies.

We therefore conducted this work first to determine the prevalence of SIBO in a cohort of patients with IBS, and second to determine whether there is a correlation between hydrogen or methane levels on breath test and the severity of IBS.

## Methods

### Study population

We screened all patients referred to our functional digestive exploration unit to perform the glucose breath test (GBT) for the diagnosis of SIBO. Patients were included prospectively from January 1, 2021 to January 1, 2022. Inclusion criteria were age >18 years and a confirmed diagnosis of IBS according to the Rome IV and III criteria, depending on the date of diagnosis. The reliability of the diagnosis was ensured by checking the clinical record of each patient. They were excluded in case of recent administration of antibiotics or probiotics (<4 weeks), if they had not respected the fasting instructions related to the test (12‐h fasting), and if they had received a transit modulator in the 7 days preceding the test. The use of proton pump inhibitors (PPIs) was not an exclusion criterion. Patients with a history of surgery were also excluded.

### Clinical evaluation

Before performing the breath test, each patient completed a self‐reported questionnaire on the severity of symptoms. To assess the severity of IBS—our primary endpoint—we used the irritable bowel severity scoring system (IBS‐SSS), which includes symptom‐focused questions, giving values ranging from 0 to 500. The first secondary endpoint, namely the quality of life, was assessed by the Gastrointestinal Quality of Life Index (GIQLI) score. This is a digestive quality‐of‐life (QoL) score consisting of 36 items covering symptoms, physical status, emotions, social problems, and the effect of medical treatments. The values obtained at the end of the questionnaire are between 0 and 144. A higher score indicates a better quality of life. The Hospital Anxiety and Depression (HAD) scale was used to assess impact on mood. This scale is used to screen for anxiety and depressive disorders through the evaluation of 14 items rated from 0 to 3. Seven questions relate to anxiety (total A) and seven others to the depressive dimension (total D), thus making it possible to obtain two results, which will then be added together (maximum rating for each score = 21).

Patients were classified into three different groups based on their dominant phenotype. This was determined by the Bristol Scale. The following categories were defined: diarrheal phenotype (IBS‐D), constipated phenotype (IBS‐C), and mixed phenotype (IBS‐M).

In the constipated group (IBS‐C), the severity of constipation was assessed by the KESS score (Knowles, Eccersley, Scott Symptom Score)^10^ with the aim of looking for a correlation with the level of methane production. This score includes a total of 12 items relating to the length of time the constipation has been present, the use of laxatives (± suppository ± enemas), the use of digital maneuvers, and the frequency of stools. A constipation is retained for a score of 10/39.

### Glucose breath test: Protocol and interpretation

Prior to the test, patients were required to follow a pre‐test preparation, which included a low‐carbohydrate diet the day before the test and a 12‐h fasting period. No smoking or exercise was allowed for at least 2 h prior to the test. Each of these measures was designed to limit the basal level of hydrogen.

An initial measurement of the gases (hydrogen and methane) was performed, and then the patients were given 50 g of glucose diluted in 250 ml of water. Every 15 min, the levels of hydrogen and methane exhaled by patients were measured, for a total period of 3 h.

During the test, and every 15 min, each patients reported the level of discomfort, bloating, and pain they experienced using a visual analog scale (VAS) with values ranging from 0 to 10.

**Figure 1:**
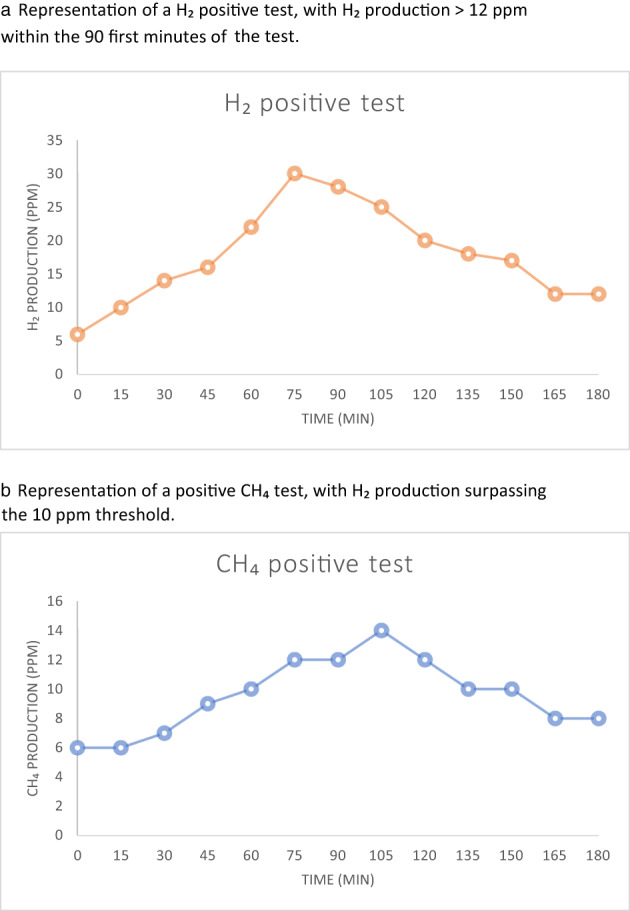
Examples of positive glucose breath tests. (a) Representation of a H_2_ positive test, with H_2_ production >12 ppm within the first 90 min of the test. (b) Representation of a positive CH_4_ test, with H_2_ production surpassing the 10 ppm threshold.

In our study, a test was considered positive for hydrogen if a rise of >12 ppm from baseline was observed within the first 90 min of the test. For methane, according to the European and American recommendations, a value >10 ppm at any time during the test was considered positive. Figure 1 shows an example of a positive breath test for H_2_ (a) and another one for CH_4_ (b).

### Statistics

Patients with baseline values >20 ppm for hydrogen were excluded from analyses because of the high suspicion of noncompliance with pre‐test instructions.

In patients tested positive for SIBO, a Pearson test was used to assess the degree of correlation between the production of each gas (H_2_, CH_4_, and H_2_ + CH_4_) and the various clinical scores (IBS‐SSS, GIQLI score, HAD scale, and VAS during testing).

A Kruskal–Wallis test was applied to compare the three subgroups of patients (IBS‐C, IBS‐M, IBS‐D) with respect to the clinical scores (IBS‐SSS, GIQLI score, HAD scale).

On the other hand, a Wilcoxon signed‐rank test was performed to carry out a comparison between the median values of the clinical scores of patients with a positive test and those with a negative test.

We decided to investigate the correlation between methane and hydrogen production and clinical severity of IBS only in patients who tested positive for SIBO. The control patients (tested negative) had by definition methane and hydrogen production below the thresholds for the diagnosis of SIBO.

Morevoer, for the statistical analysis, we decided to work with median values of gas production and clinical scores because of their very heterogeneous distribution, including the potential existence of extreme values (especially concerning methane and hydrogen production).

### Ethics

All patients included in this study provided written and signed consent. As the study protocol was based on the completion of a questionnaire, patients were not exposed to any additional risk. The GBT for which they were referred was performed according to the routine practices of our center. The study was conducted with the approval of the Clinical Research Department (DRCI) of the Nice University Hospital. The trial was also declared to the national registry.

## Results

### Prevalence of SIBO in IBS

Two‐hundred and fifty patients with IBS provided consent for inclusion in the study. Three were excluded from the analysis because of a basal H_2_ value >20 ppm. Two of them tested positive both for methane and hydrogen production, with very high basal values. The demographic characteristics of the population are given in Table 1.

**Table 1:** Characteristics of the study population

Parameters	Value
Age, years (min.–max.)	49.8 (16–85)
Gender	
Male (*n*) (%)	56 (22.67)
Female (*n*) (%)	191 (77.33)
IBS	
Constipation (*n*) (%)	82 (33.19%)
Diarrhea (*n*) (%)	100 (40.49%)
Alternating (*n*) (%)	65 (26.32%)
Bloating (*n*) (%)	229 (92.71%)
Starting age in years (mean) [range]	39.5 [9–78.5]
Duration of disease in years (mean) [range]	9.1 [0.5–56]
BMI (mean)	23.1 ± 5.1
IBS‐SSS (median) [range]	268 [25–496]
GIQLI score (median) [range]	77.15 [25–133]
HAD scale	
Total (median) [range]	18.06 [0–37]
Depression (median) [range]	8.05 [0–20]
Anxiety (median) [range]	10.01 [0–21]

BMI, body mass index (kg/m^2^); GIQLI, Gastrointestinal Quality of Life Index; HAD, Hospital Anxiety and Depression; IBS‐SSS, irritable bowel severity scoring system.

In this cohort, the prevalence of SIBO was determined to be 36.4% (90/247). More precisely, 24 patients (9.7%) had a positive test due to a significant elevation of hydrogen, while 66 (26.7%) had an elevated methane level. None of the patients included in the analysis tested positive for both gases.

Regarding the proportion of patients on PPIs, there was no significant difference between the group of patients with a positive test (17.9%) and those with a negative test (27.5%) (*P* = 0.10).

### Gas production according to dominant phenotype

Of the 90 patients tested positive, 36 (40%) had a predominantly constipated phenotype (IBS‐C), 29 (32.22%) a predominantly diarrheal phenotype (IBS‐D), and 25 (27.78%) an alternating phenotype (IBS‐M).

**Figure 2:**
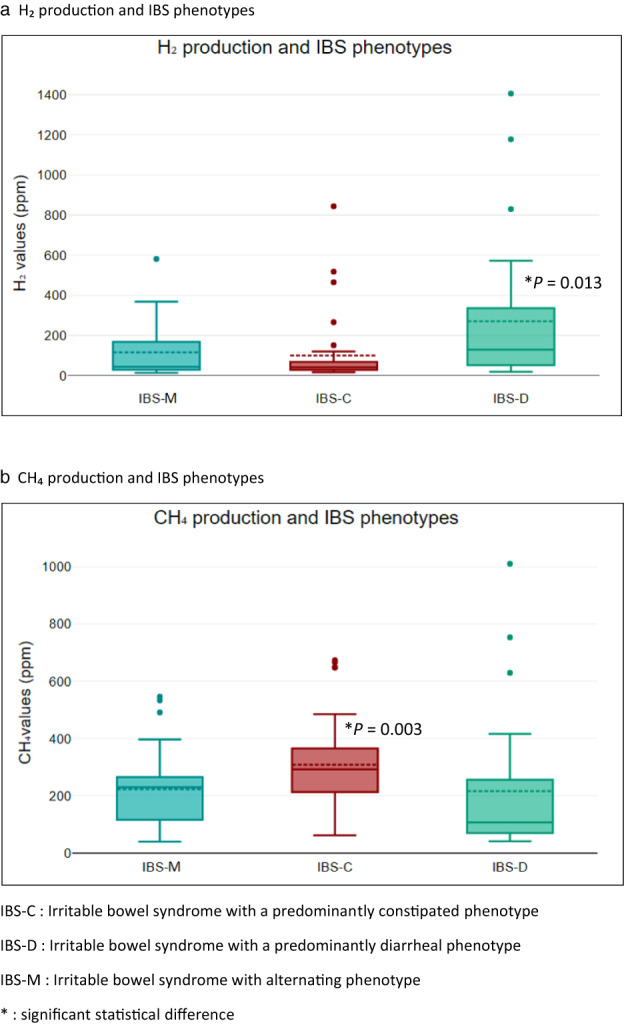
Gas production according to irritable bowel syndrome (IBS) phenotypes. (a) H_2_ production and IBS phenotypes. (b) CH_4_ production and IBS phenotypes. IBS‐C, irritable bowel syndrome with a predominantly constipated phenotype; IBS‐D, irritable bowel syndrome with a predominantly diarrheal phenotype; IBS‐M, irritable bowel syndrome with alternating phenotype. *Significant statistical difference.

The sum of methane values measured during the respiratory tests was significantly higher in patients with a predominantly constipated phenotype (median [range]: SII‐C 292.50 ppm [62–673] *vs* SII‐D 107 ppm [41–1010] and SII‐A 44 ppm [100–811], *P* = 0.00). Hydrogen production was significantly higher in patients with a diarrheal phenotype (median [range]: SII‐C 41.50 ppm [17–844] *vs* SII‐D 129 ppm [19–1406] and SII‐A 40 ppm [14–581], *P* = 0.01). The sum of hydrogen and methane values measured during the tests did not differ statistically within the three sub‐phenotypes, *P* = 0.17. Figure 2 shows the median values of hydrogen and methane production according to the three sub‐phenotypes.

The three phenotype subgroups did not differ statistically with respect to the IBS‐SSS (*P* = 0.78), the GIQLI score (*P* = 0.29), and the HAD score (total A *P* = 0.65, total D *P* = 0.62). Table 2 shows the clinical scores for each phenotype.

**Table 2:** Clinical scores according to IBS phenotypes

Clinical scores	IBS‐C	IBS‐D	IBS‐M	*P*‐value
IBS‐SSS (median) [range]	240 [25–490]	295 [60–420]	230 [55–470]	0.782
GIQLI score (median) [range]	81 [44–112]	73 [25–113]	78 [53–122]	0.293
HAD total (median) [range]	17 [7–32]	17 [6–32]	14 [9–31]	0.455
HAD Anxiety (median) [range]	9.5 [4–19]	10.5 [3–18]	6 [2–17]	0.653
HAD Depression (median) [range]	8 [3–16]	8.5 [3–19]	8 [4–16]	0.622

GIQLI, Gastrointestinal Quality of Life Index; HAD, Hospital Anxiety and Depression; IBS‐SSS, irritable bowel severity scoring system.

### Gas production and severity of irritable bowel syndrome

**Figure 3:**
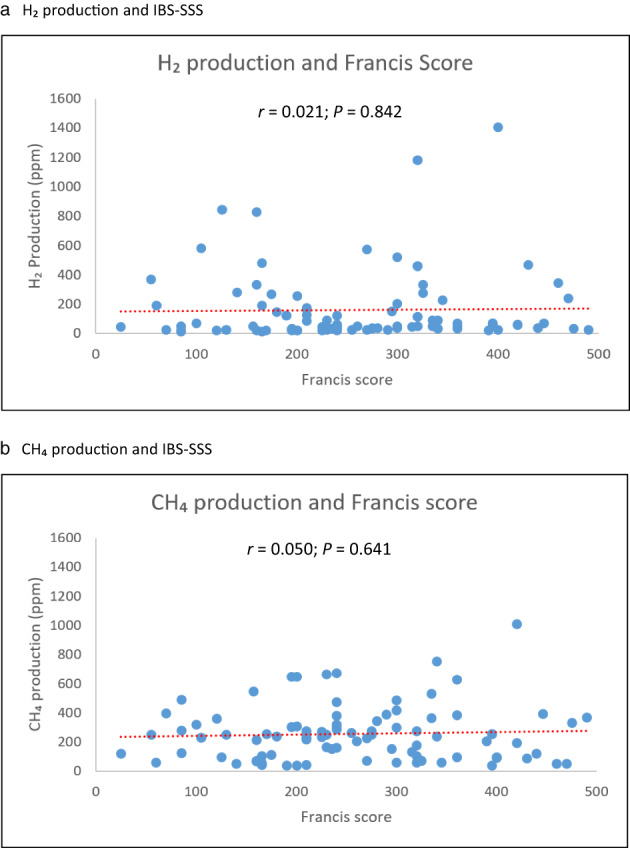
Gas production and IBS‐SSS. (a) H_2_ production and IBS‐SSS. (b) CH_4_ production and IBS‐SSS.

IBS severity as assessed by IBS‐SSS was not correlated with either total methane production (sum of methane values measured during testing) (*r* = 0.05; *P* = 0.64) or hydrogen production (sum of hydrogen values measured during testing) (*r* = 0.02; *P* = 0.84), Figure 3a,b. The median values of IBS‐SSS did not differ statistically between the group of patients with SIBO (240 [55–490]) and those with a negative test (275 [100–496]), *P* = 0.316 (Table 3).

**Table 3:** Clinical scores according to the result of glucose breath test (GBT)

Clinical scores	Positive GBT	Negative GBT	*P*‐value
IBS‐SSS (median) [range]	240 [55–490]	275 [100–496]	0.316
GIQLI score (median) [range]	78 [44–114]	71 [30–108]	0.273
HAD total (median) [range]	17 [7–32]	18 [6–35]	0.919
HAD Anxiety (median) [range]	8.5 [4–19]	10 [4–20]	0.679
HAD Depression (median) [range]	7 [2–17]	9 [1–20]	0.582

GIQLI, Gastrointestinal Quality of Life Index; HAD, Hospital Anxiety and Depression; IBS‐SSS, irritable bowel severity scoring system.

Regarding methane specifically, its production was not correlated with the quality of life (GIQLI score; *r* = 0.00; *P* = 0.99), with the HAD D depression scale (*r* = −0.15; *P* = 0.06), or with the HAD A anxiety scale (*r* = −0.192 *P* = 0.07).

In contrast, hydrogen production was inversely correlated with the quality of life (GIQLI score; *r* = −0.24; *P* = 0.03) and significantly correlated with the HAD scale (total HAD; *r* = 0.22; *P* = 0.03). This correlation was significant on depression (HAD D; *r* = 0.22; *P* = 0.04) but not significant on anxiety (HAD A; *r* = 0.17; *P* = 0.12).

**Figure 4:**
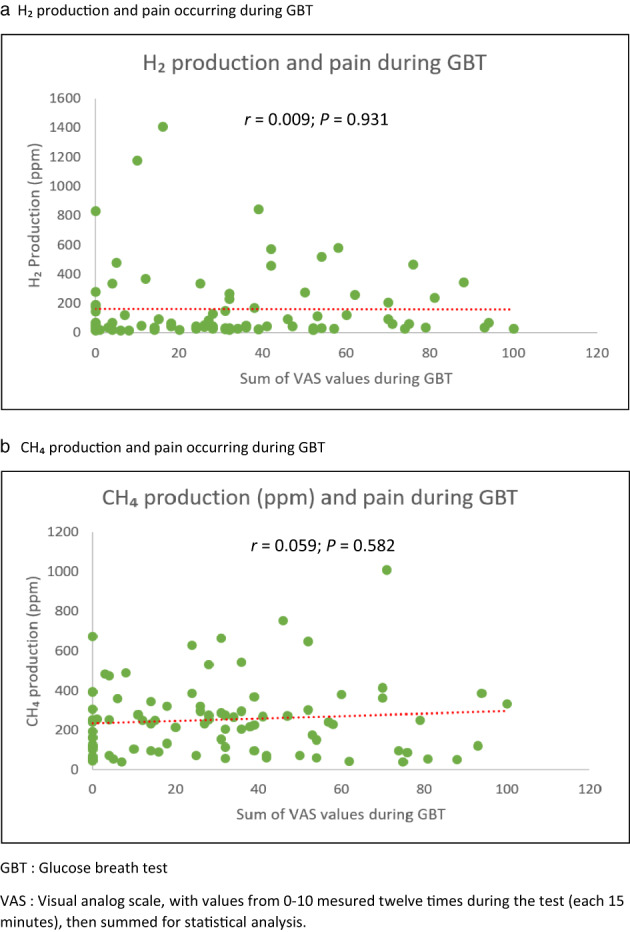
Gas production and pain/incomfort reported during glucose breath test (GBT). (a) H_2_ production and pain occurring during GBT. (b) CH_4_ production and pain occurring during GBT. VAS, visual analog scale, with values from 0 to 10 mesured 12 times during the test (each 15 min), and then summed for statistical analysis.

**Figure 5:**
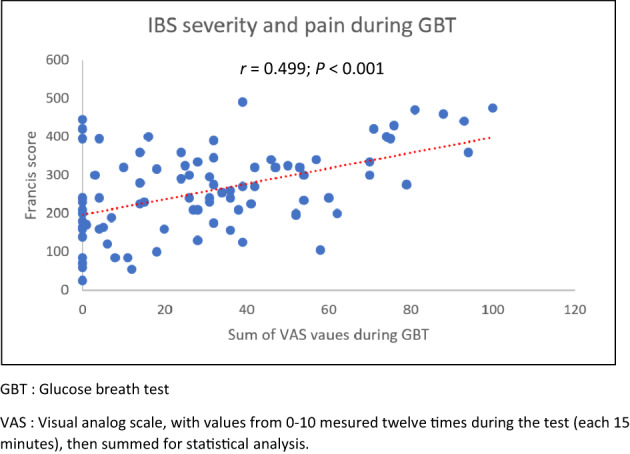
Correlation between IBS severity and pain during GBT. VAS, visual analog scale, with values from 0 to 10 mesured 12 times during the test (each 15 min), and then summed for statistical analysis.

The level of pain and discomfort experienced during the breath test (VAS) did not correlate with the level of gas produced during the same test, whether it was hydrogen (*r* = −0.01, *P* = 0.931) (Fig. 4a), methane (*r* = −0.09, *P* = 0.40) (Fig. 4b), or the sum of both (*r* = 0.06, *P* = 0.58). On the other hand, there was a significant correlation between the VAS reported during the glucose breath tests and the level of severity of the IBS (*r* = 0.50, *P* < 0.00) (Fig. 5). Similarly, the quality of life was negatively correlated with the level of VAS (*r* = −0.28, *P* = 0.01).

The severity of constipation (KESS score) was not correlated with either the level of methane produced (*r* = −0.11, *P* = 0.43) or the level of hydrogen (*r* = −0.02, *P* = 0.91).

## Discussion

In our study, the prevalence of SIBO in IBS was found to be 36.8%. In this population of patients, the severity of IBS was not correlated with the measured gas levels in the breath test, whether it was H_2_, CH_4_, or the sum of both. CH_4_ levels were significantly greater in patients with a constipated phenotype, while H_2_ showed a higher degree in patients with a diarrheal phenotype. The level of pain and discomfort experienced during GBT was not correlated with the amount of methane and/or hydrogen measured during the test, but was significantly associated with IBS severity. Finally, H_2_ production could be associated with altered mood, with a predominance on depression.

In this study, the prevalence of SIBO in IBS was determined to be 36.8%. In a systematic review^2^ of 28 studies investigating the prevalence of SIBO in IBS, values ranging from 4%^5^ to 78%^6^ were reported. Such variability can be explained by the use of different diagnostic methods: culture of colonic fluid (5 studies), lactulose breath tests (11 studies), and glucose breath tests (12 studies). However, the choice of the technique, and more specifically that of the substrate, is fundamental given the variations in performance observed in the literature. In our study, we opted for breath tests because of their accessibility and excellent acceptability. Regarding the substrates, we chose glucose because it would provide better results than lactulose regarding the sensitivity (62% *vs* 52%) and the diagnostic accuracy of breath tests in SIBO (72% *vs* 55%).^2^, ^11^

One of the strengths of our study lies in the choice of the thresholds used for the interpretation of breath tests. Indeed, regardless the European^7^ and American^8^ consensus conferences, the question of optimal thresholds for the diagnosis of SIBO remains debated. A recent meta‐analysis specifically evaluated the diagnostic performance of GBT in this respect. For this purpose, the sensitivity and specificity values of several thresholds were compared with those of small‐bowel liquid culture, which can be considered as a reference test. The results of this study support the American and European recommendations in their choice of a 10‐ppm threshold for the positivity of GBTs for CH_4_. Concerning H_2_, this work showed that a threshold of 12 ppm should be preferred because it provides better sensitivity and specificity than the 20‐ppm threshold recommended by the American Society of Gastroenterology (sensitivity 61.7% *vs* 47.3%; specificity 86% *vs* 80.9%; AUC‐SROC 0.79 ± 0.07 *vs* 0.70 ± 0.23).^9^ In our study, the positivity criteria for H_2_ were expected to be reached within the first 90 min of testing. This cut‐off is recommended by the American consensus in order to limit the risk of false positives that would result from colonic fermentation in individuals with rapid orocaecal transit time. This parameter is generally a limitation regarding the reliability of breath tests in the diagnosis of SIBO, especially when lactulose is used.^12^, ^13^ For CH_4_, it is recommended not to use the 90‐min cut‐off time when interpreting the tests. Note that the results of a wide population‐based study suggest that basal CH_4_ values ≥5 and ≥10 ppm could predict excessive CH_4_ production with a specificity of 99.7% and 100% and a sensitivity of 96.1% and 86.4%, respectively.^14^

Our results suggest that SIBO is common during IBS but does not worsen its severity. The levels of IBS severity were statistically comparable between SIBO‐positive and control patients. Furthermore, the gas levels measured were not correlated with the severity of IBS in patients tested positive for SIBO. The few studies that have investigated this question have generally been based on a comparison of measured gas levels, with VAS rated from 0 to 10.^4^, ^15^, ^16^ Although most of these studies also confirm the absence of correlation between the gas levels produced and the severity of IBS symptoms, our study has the specific advantage of using a more robust and global assessment tool, namely the IBS‐SSS. Our work underlines the preponderant role of intestinal hyperalgesia. Indeed, in our cohort, the patients who reported the highest levels of pain and discomfort during the tests were also those with the highest IBS severity scores. This correlation was also observed with the QoL score. On the other hand, we also found a lack of correlation between the levels of CH_4_ or H_2_ measured during the test and the level of discomfort felt by the patients during the test. Thus, individual susceptibility to pain would have a greater impact than gas production on IBS severity.

Several authors have highlighted a specific role of CH_4_ in the development of symptoms associated with constipation.^3^, ^17^ CH_4_ is thought to act as a neurotransmitter in the digestive tract, slowing down the intestinal transit. This association between CH_4_ production and constipation has been confirmed by several studies, even though the data diverge concerning the correlation between the levels of CH_4_ measured and the severity of constipation.^4^, ^16^ In our study, we also found a significant association between CH_4_ levels during breath tests and the development of a predominantly constipated IBS phenotype. However, the severity of constipation was not found to be correlated with the levels of CH_4_ measured, as confirmed by a recent study.^16^ On the other hand, we observed a significantly higher H_2_ production and number of positive H_2_ tests in patients with a diarrheal phenotype. This has also been reported in a recent work.^18^

There is debate about the impact of PPIs on the development of SIBO. Indeed, from a pathophysiological point of view, PPI‐induced hypochlorhydria could promote intestinal bacterial proliferation.^19^ A meta‐analysis published in 2018 based on data from 19 studies confirmed a moderate increase in the risk of SIBO in patients on PPIs (OR 1.71, 95% confidence interval 1.20–2.43).^20^ However, some studies, including a very recent one, do not confirm this excess risk of SIBO under PPI.^21^, ^22^ Based on these studies, we have chosen not to exclude patients on PPIs from the analysis. This approach is supported by our results, which confirm the absence of any statistical difference between control and SIBO patients with regard to PPI use (IBS with SIBO 17.9%, IBS without SIBO 27.5%, *P* = 0.10).

H_2_ produced by the gut microbiota is partly consumed by certain populations of gut flora to produce CH_4_ (40% of H_2_‐consuming bacteria) and H_2_ sulfide (55% of H_2_‐consuming bacteria).^3^, ^23^ This contributes to a decrease in the levels of H_2_ measured during breath tests, sometimes resulting in false negative tests for H_2_. It is therefore essential to systematically measure CH_4_ production when performing breath tests for the diagnosis of SIBO. This view is supported by the high prevalence of positive CH_4_ tests found in our cohort. Because the methanogenic flora includes archaea and some bacterial populations of the Clostridium and Bacteroides types, the terminology of SIBO might seem less appropriate when a test is positive for methane. The term IMO (intestinal methanogen overgrowth) has been proposed by the American College of Gastroenterology for these cases.

It has been demonstrated that the intestinal microbiota is involved in mood disorders, as clear differences have been found between the intestinal microbiota of healthy subjects and those of patients with psychiatric disorders^24^, ^25^, ^26^, ^27^ and autism.^28^ In the present study, the results of anxiety and depression scales were statistically comparable between the positive and control patients. However, the effect of SIBO on mood might be underestimated in this context because of the classically high prevalence of neuropsychiatric pathologies in IBS. Nevertheless, our study shows a correlation between the levels of H_2_ produced and mood disorders, with a predominance on depression. As H_2_ is mainly produced by Bacteroides bacteria, it is interesting to put our results in perspective with those of a study which observed an over‐representation of this family of bacteria in the intestinal microbiota of patients suffering from depression.^25^ However, these results must be considered with certain reservations because other studies have found conflicting results.^24^ As research progresses, the microbiota now appears as a potential therapeutic target in the management of neuropsychiatric pathologies.^26^, ^29^, ^30^ The correlation between H_2_ production and the HAD scale could also partly explain the negative correlation observed between H_2_ levels and the QoL score (GIQLI score), as the latter gives high consideration to psychologic items.

The results of our study question the utility of screening for SIBO during IBS. Indeed, patients who tested positive had a severity score of IBS statistically comparable to that of control patients. However, Pimentel *et al*. have demonstrated an improvement in IBS symptoms when SIBO is eradicated by oral antibiotic therapy.^6^ The interest of diagnosing SIBO in this population would therefore be to determine which patients are likely to benefit from antibiotic therapy, particularly in case of failure of the usual treatments.

Given the subjective nature of IBS symptom evaluation, our results could be affected by an assessement bias, which we attempted to limit by using robust questionnaires. On the other hand, as all patients were referred to perform breath tests for the detection of SIBO despite an established diagnosis of IBS, there could be a recruitment bias whose impact is difficult to evaluate. Indeed, as the reasons for testing are not recorded, it is impossible to know, for example, whether most patients were referred because of treatment inefficiency, in order to detect SIBO, which could provide new therapeutic options. Nevertheless, as our population is globally comparable to other IBS cohorts in terms of demographic characteristics and clinical severity scores, this recruitment bias seems to have only a limited impact. Lastly, our results are based on gas levels measured during the breath test as indirect indicators of intestinal gas volumes. The correlation between the two could be incomplete and may affect our results.

## Conclusion

At the end of our study, we conclude that gas production related to intestinal microbial overgrowth does not worsen the severity of symptoms in patients suffering from IBS. This severity appears to be more related to an individual susceptibility to pain, underlining the importance of intestinal hyperalgesia in the perception of symptoms during IBS. Therefore, we feel that reduction of gas levels in breath tests should not be considered as a relevant clinical goal in these patients. Given this, we also question the relevance of repeated antibiotic therapy and repeated breath tests in patients with SIBO and IBS. Improvement of symptoms after eradication of SIBO should be investigated in further randomized studies.
